# Colchicine-Induced Polyploidization Influences the Morphological, Physiological, and Biochemical Characteristics of *Cyclocarya paliurus*

**DOI:** 10.3390/plants14172778

**Published:** 2025-09-05

**Authors:** Guoliang Bian, Yan Yi, Ziqi Song, Yanmeng Huang, Qianxing Mao, Jian Qin, Xulan Shang

**Affiliations:** 1 College of Forestry and Grassland, Nanjing Forestry University, Nanjing 210037, China; bianguoliang@njfu.edu.cn (G.B.); 19852851100@163.com (Y.Y.); songziqi@issas.ac.cn (Z.S.); huangyanmengzzz@163.com (Y.H.); 19870641625@163.com (Q.M.); qinjian320@163.com (J.Q.); 2 National Key Laboratory for the Development and Utilization of Forest Food Resources, Nanjing Forestry University, Nanjing 210037, China; 3 Co-Innovation Center for the Sustainable Forestry in Southern China, Nanjing 210037, China

**Keywords:** *Cyclocarya paliurus*, Polyploidization, Colchicine, induced tetraploid, induced octoploid

## Abstract

*Cyclocarya paliurus* (Batal.) Iljinskaja is a rare and multifunctional tree species endemic to China. This study aimed to establish a feasible method for polyploid induction in *C. paliurus* with colchicine treatment, and the obtained polyploid plants were identified and characterized. In this study, natural diploid and tetraploid *C. paliurus* seedlings were treated with different concentrations and durations of colchicine to induce polyploidization. The results indicated that a colchicine concentration of 0.4% for 4 d was the most suitable induction protocol, respectively. Compared with diploid and tetraploid control plants, the induced polyploid plants were shorter and thinner. The number of compound leaves in induced polyploids were fewer, and the compound leaf areas became smaller. The stomatal density of induced polyploids decreased, but the stomata became larger and wider, with an increased number of chloroplasts in the guard cells. The induced polyploids exhibited higher levels of carotenoid, and the contents of soluble sugar, soluble starch, and soluble protein were higher than those of controls. The polyploid plants exhibited an increase in the contents of growth-inhibiting hormones (JA) and a decrease in those of growth-promoting hormones (IAA, GA_3_, BR). In summary, the induced polyploids with a dwarfing effect would provide superior germplasm resources for leaf-harvesting plantation. As *C. paliurus* is endemic to China, our results have a rather local effect. Overall, the established polyploid induction method of *C. paliurus* will provide technical support for creating superior *C. paliurus* germplasm resources and subsequent plant breeding research.

## 1. Introduction

*Cyclocarya paliurus* (Batal.) Iljinskaja is the sole species within its genus, prevalent in the subtropical mountainous regions of China [1]. This species is renowned for its versatility, and its wood quality is comparable to that of *Juglans nigra*, *Juglans mandshurica*, and *Engelhardtia roxburghiana* [2,3]. Moreover, because the leaves are abundant in bioactive compounds, including triterpenoids, polysaccharides, flavonoids, and polyphenols, the leaves of *C. paliurus* have been utilized in herbal teas and Chinese medicinal practices [4,5]. The National Health and Family Planning Commission of China granted approval for the leaves of *C. paliurus* to be a new food raw material in 2013 [6]. Due to their high medicinal benefits, there was an extensive market demand for the leaves, leading to the depletion of the natural resources of *C. paliurus*. Consequently, developing *C. paliurus* plantations is the optimal strategy for producing leaves and protecting natural resources. However, most *C. paliurus* plantations are being established on the mountains and hilly areas of the sub-tropical region of China, where the growing environment is poor; thus, it is essential to obtain new germplasm resources with superior environmental adaptation for the healthy development of the *C. paliurus* industry.

Polyploidization is pivotal in plant evolution and breeding [7,8]. Ploidy breeding, in particular, offers the potential to acquire superior features, such as greater adaptability and resistance [9]. Polyploidization, the doubling of a species’ entire chromosome set to derive a new plant [10], significantly alters the plant’s physiological and chemical characteristics. Artificial induction of polyploidy is achieved through chemical (the disruption of the plant cell cycle via chemical reagents) and physical (temperature, centrifugation, ultrasound, and radiation) methods [11]; among them, chemical methods are the most prevalent and efficacious approaches. Colchicine is the most frequently employed and efficacious reagent of chemical induction, and it has been effectively employed to induce polyploidy in diverse woody plants, such as *Rhododendron fortunei* [12], *Ficus carica* [13], and *Ziziphus jujuba* [14]. The low affinity of colchicine for plant microtubules requires a higher concentration to achieve chromosomal doubling, leading to increased damage to plant components. Moreover, colchicine may induce plant infertility, aberrant growth, chromosomal loss or rearrangement, and genetic abnormalities [8].

Owing to the short history of artificial cultivation of *C. paliurus*, the application of colchicine for polyploidization of *C. paliurus* remains unreported. Thus, the aims of this study were to devise a preliminary polyploidy induction protocol applicable to *C. paliurus*, to evaluate the effect of polyploidization on leaf characteristics, and to explore the mechanism from the perspective of physiological, anatomical, and phytochemical traits. The exploration of the artificial polyploidization of *C. paliurus* will provide technical support for creating superior germplasm resources of *C. paliurus* and subsequent plant breeding research.

## 2. Results

### 2.1. Polyploidy Induction Rate

During a two-year period, 720 seedlings were used for polyploid induction. As shown in Table 1, *C. paliurus* seedlings treated with 0.4% colchicine for 4 d gained the highest survival rate (35.00%), octoploid induction rate (20.00%), and mixoploid induction rate (10.00%) in 2021. The survival rate, octoploid induction rate, and mixoploid induction rate of *C. paliurus* were lower under the treatment with 0.3% colchicine for different treatment times and under 0.4% colchicine for 7 d, and no seedlings survived under the treatment with 0.6% colchicine. To ensure that more induced polyploidization seedlings would be obtained, 0.4% colchicine was selected as the optimum concentration for subsequent experiments. In the second year, WF4x seedlings treated with 0.4% colchicine for 4 d obtained a 52.08% survival rate, a 39.58% octoploid induction rate, and an 8.33% mixoploid induction rate (Table 1).

In 2022, WF2x seedlings were treated with 0.4% colchicine for different durations, and the survival rate and tetraploid induction rate decreased gradually with the extension of colchicine treatment time (Table 1). The highest survival rate, tetraploid induction rate, and mixoploidy induction rate of *C. paliurus* seedlings were obtained under the treatment with 0.4% colchicine for 4 d.

### 2.2. Ploidy Determination

**Figure 1:**
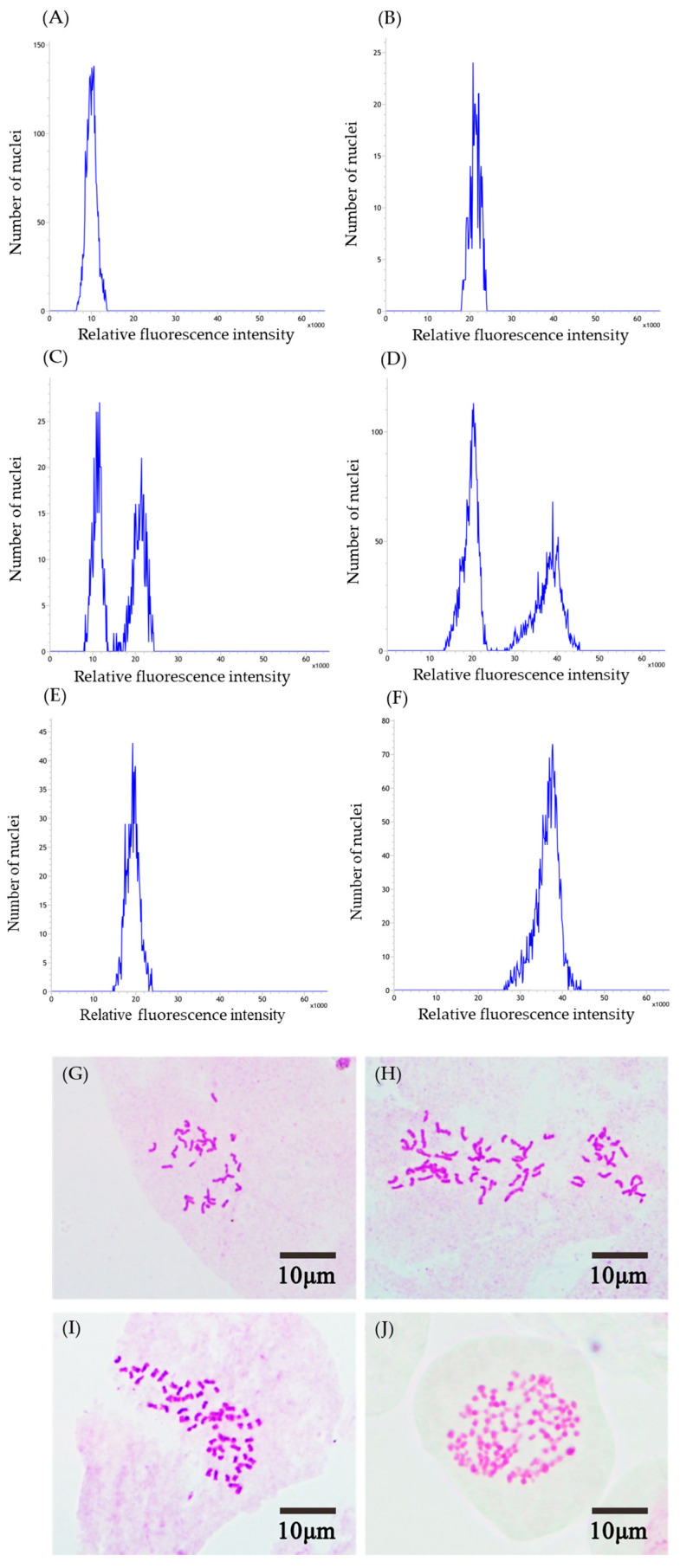
Flow cytometry analysis of *C. paliurus* (**A**) N2x, (**B**) N4x, (**C**,**D**) mixoploid, (**E**) I4x, (**F**) I8x, and chromosomes of (**G**) N2x, (**H**) I4x, (**I**) N4x, and (**J**) I8x at 1000× magnification.

Flow cytometry analysis was conducted to determine the ploidy levels in the treated seedlings. As shown in Figure 1, natural diploid (N2x) exhibited a peak at a relative fluorescence intensity of 9349 (Figure 1A), while induced tetraploid (I4x) produced a peak at 19,276 (Figure 1E). Meanwhile, the mixoploid plants induced from natural diploids simultaneously exhibited peaks at 10,766 and 20,820 (Figure 1C). Natural tetraploid (N4x) and induced octoploid (I8x) exhibited peaks at relative fluorescence intensities of 20,600 and 37,558, respectively (Figure 1B,F). Meanwhile, the mixoploid plants induced from natural tetraploids simultaneously exhibited peaks at 20,253 and 40,172 (Figure 1D). Finally, we obtained 40 induced tetraploid seedlings (I4x) and 11 mixoploid plants induced from WF2x, as well as 33 octoploid plants (14 induced from JZS4x and 19 induced from WF4x) and 17 mixoploid plants (13 induced from JZS4x and 4 induced from WF4x). The ploidy levels of the control (N2x and N4x) and induced polyploid seedlings (I4x and I8x) were also validated through chromosome counting. It was affirmed that the chromosome numbers in the induced tetraploid plants were doubled (2n = 4x = 64) (Figure 1H) compared with the natural diploid plants (2n = 2x = 32) (Figure 1G). Similarly, the chromosome numbers in the induced octoploid plants were doubled (2n = 8x = 128) (Figure 1J) compared with the natural tetraploid plants (2n = 4x = 64) (Figure 1I).

### 2.3. Comparative Analysis of Growth and Morphological Characteristics

**Figure 2:**
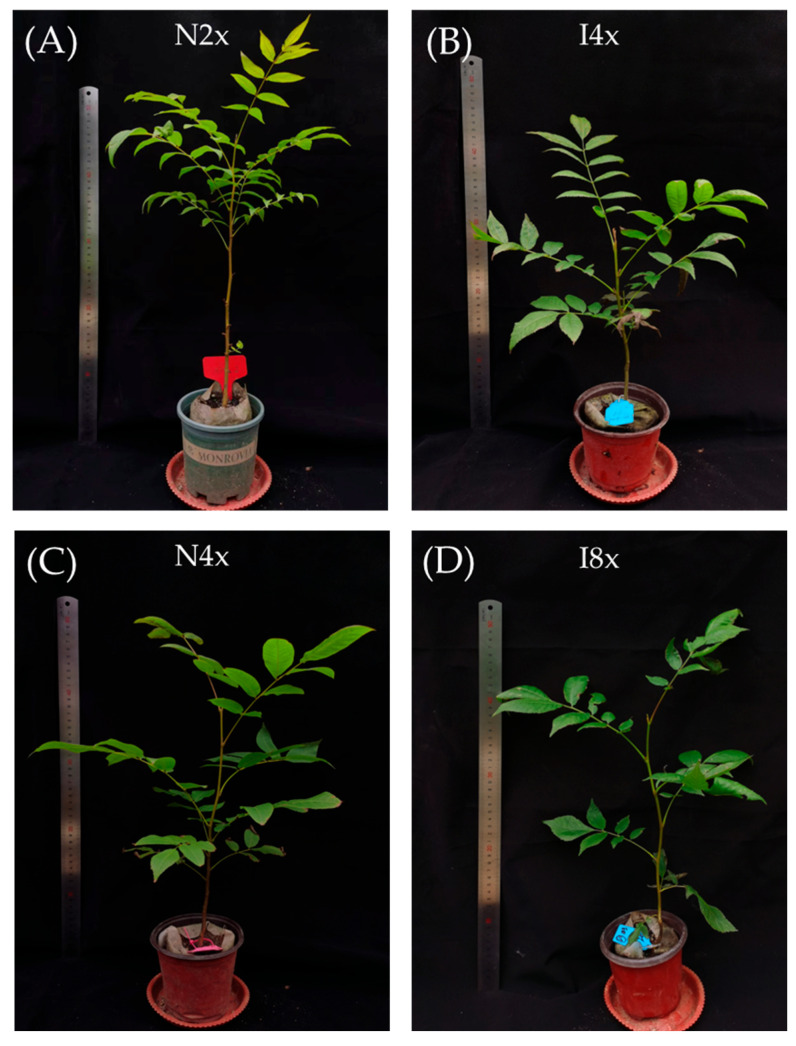
Plant morphological characteristics of *C. paliurus* seedlings at different ploidy levels: (**A**–**D**) 150-day-old control and induced plants.

After 150 days of cultivation, there were significant differences in seedling height and basal diameter between natural plants and induced plants, except for basal diameter between I4x and N2x (*p* < 0.05, Table 2, Figure 2A–D). I4x seedlings were 18.70% shorter than N2x seedlings. In comparison to the control, the basal diameters of I4x and I8x diminished by 16.78% and 18.33%, respectively.

Significant differences in morphological characteristics were noticed between control and induced polyploids, except for specific leaf weight between I4x and N2x (*p* < 0.05, Table 2). Induced polyploids had a reduced compound leaf area, fewer compound leaves, and increased terminal leaflet length. The compound leaf area of N2x was 1.22 times larger than that of I4x, while N4x exhibited a compound leaf area 1.91 times greater than that of I8x. The number of compound leaves in N2x and N4x was 1.53 times and 1.4 times greater than that of the induced polyploids, respectively. Terminal leaflet length of induced polyploids significantly increased up to 19% in I4x and 27% in I8x (*p* < 0.05). Moreover, in comparison to N4x, the specific leaf weight and terminal leaflet width rose significantly by 8.03% and 26.07%, respectively.

### 2.4. Analysis of Leaf Anatomical Structure

**Figure 3:**
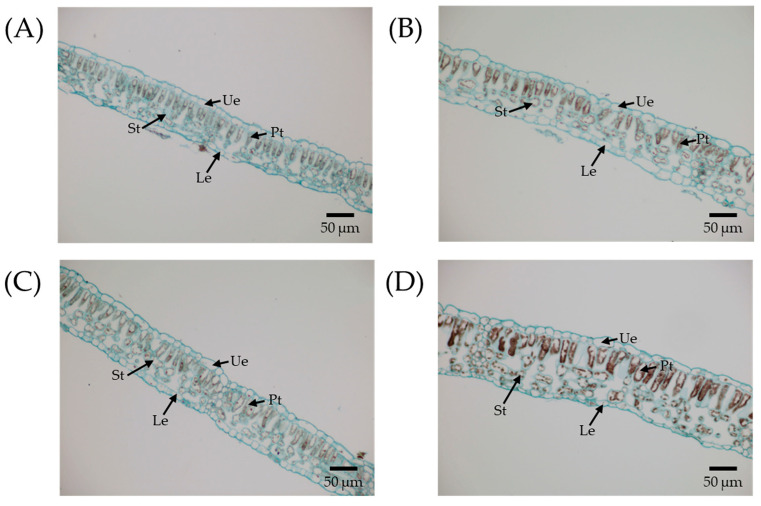
Comparison of anatomical and structural features of leaves of different ploidies of *C. paliurus* seedlings. (**A**) N2x, (**B**) I4x, (**C**) N4x, and (**D**) I8x. Ue, upper epidermis; Le, lower epidermis; Pt, palisade tissue; St, spongy tissue.

Comparative analysis using paraffin sections showed that the leaflet became thicker with octoploidization. Significant differences were observed in leaflet thickness, palisade tissue thickness, and spongy tissue thickness between N4x and I8x (*p* < 0.05, Table 3, Figure 3). Specifically, the leaflet thickness of I8x plants increased by 19.02% compared to N4x. In I8x plants, the thickness of the palisade tissue and spongy tissue increased by 18.88% and 37.60%, respectively, in contrast to N4x control plants. In addition, there were no significant differences in epidermal thickness and the ratio of palisade tissue to spongy tissue between natural plants and induced plants (*p* > 0.05).

### 2.5. Analysis of Stomatal Characteristics

Significant differences were observed in the dimensions of stomata, density of stomata, and number of chloroplasts between natural plants and induced plants (*p* < 0.05, Table 4). Stomatal length and width in induced polyploids were larger than those in diploid and tetraploid control plants, respectively. The stomatal length and width of I8x plants were 16.75% and 22.09% larger than those of N4x plants, respectively. Moreover, the mean number of chloroplasts in I4x and I8x was 1.47 times and 1.31 times larger than that of the control plant.

**Figure 4:**
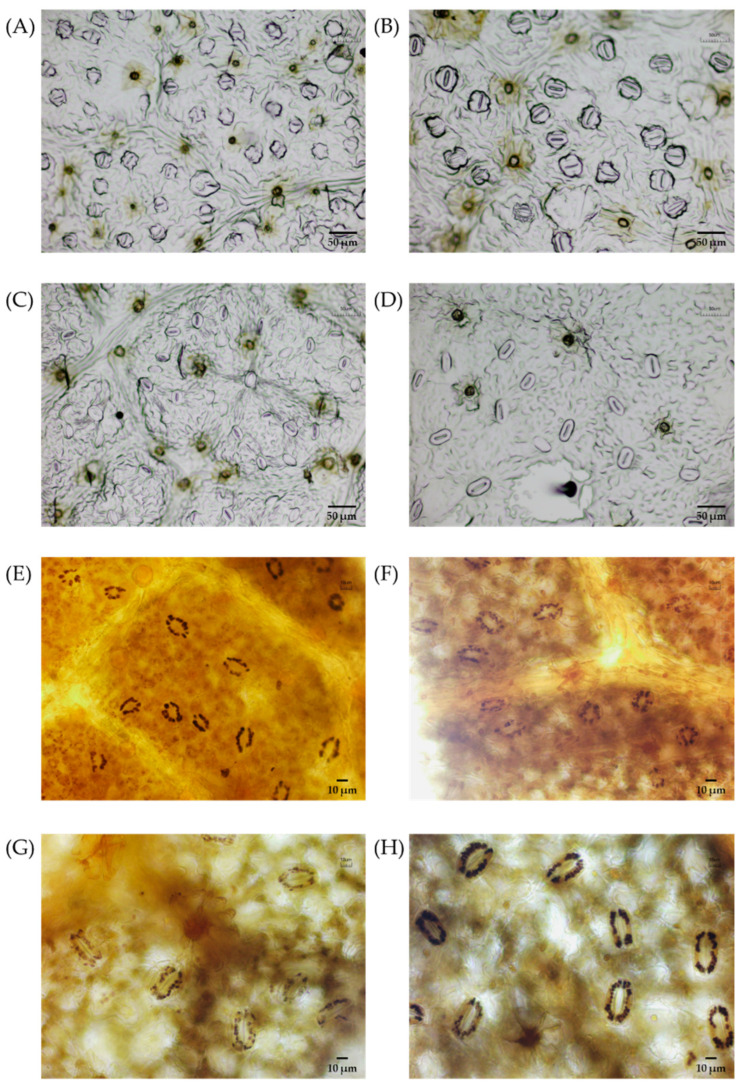
Comparison of stomatal characteristics of different ploidy levels of *C. paliurus* seedlings. (**A**) N2x stomata, (**B**) I4x stomata, (**C**) N4x stomata, and (**D**) I8x stomata; (**E**) chloroplasts in the N2x stomata, (**F**) chloroplasts in the I4x stomata, (**G**) chloroplasts in the N4x stomata, and (**H**) chloroplasts in the I8x stomata.

However, the stomatal densities in control plants were significantly higher than those in induced polyploid plants (*p* < 0.05, Table 4, Figure 4). Specifically, the stomatal density of N2x was 2.73 times higher than that of I4x, while N4x exhibited a stomatal density 2.54 times greater than that of I8x.

### 2.6. Analysis of Photosynthetic Pigments in Leaves

**Figure 5:**
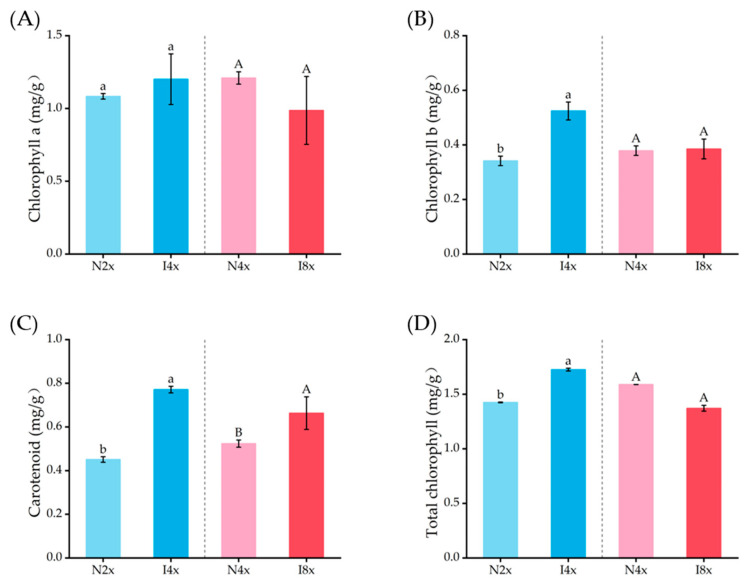
Comparison of photosynthetic pigment content including chlorophyll a (**A**), chlorophyll b (**B**), carotenoid (**C**), and total chlorophyll content (**D**) in leaves of different ploidies of *C. paliurus*. An independent-samples t-test was used to test the significance (*p* ≤ 0.05). Lowercase letters indicate significance between N2x and I4x (*p* ≤ 0.05), and uppercase letters indicate significance between N4x and I8x (*p* ≤ 0.05).

Induced polyploidization significantly increased the content of carotenoids in the leaves (*p* < 0.05, Figure 4). Specifically, there was a 71.00% increase in I4x compared to N2x and a 26.64% increase in I8x compared to N4x. With respect to the content of chlorophyll b and total chlorophyll, in I4x, they were 1.54 times and 1.21 times higher than those of N2x, whereas I8x and N4x showed no significant difference (*p* > 0.05, Figure 5).

### 2.7. Analysis of Primary Metabolites in Leaves

**Figure 6:**
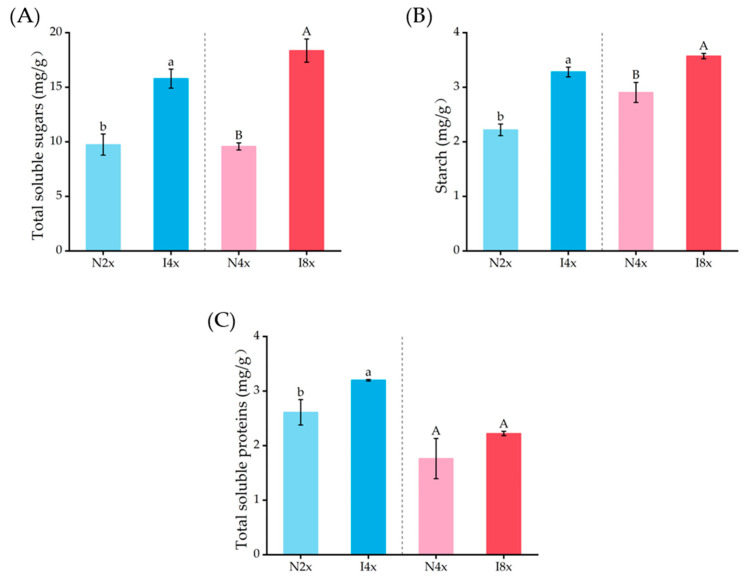
Comparison of primary metabolite contents, including total soluble sugars (**A**), starch (**B**), and total soluble protein (**C**), in leaves of different ploidies of *C. paliurus*. An independent-samples t-test was used to test the significance (*p* ≤ 0.05). Lowercase letters indicate significance between N2x and I4x (*p* ≤ 0.05), and uppercase letters indicate significance between N4x and I8x (*p* ≤ 0.05).

Induced polyploidization significantly increased the contents of starch, soluble sugar, and soluble protein in leaves (*p* < 0.05, Figure 6). The concentration of soluble sugar, starch, and soluble protein in I4x was 1.48, 1.62, and 1.23 times greater than that of N2x, respectively. The concentration of soluble sugar, starch, and soluble protein in I8x was 1.23, 1.92, and 1.26 times greater than that of N4x, respectively.

### 2.8. Analysis of Endogenous Hormones in Leaves

**Figure 7:**
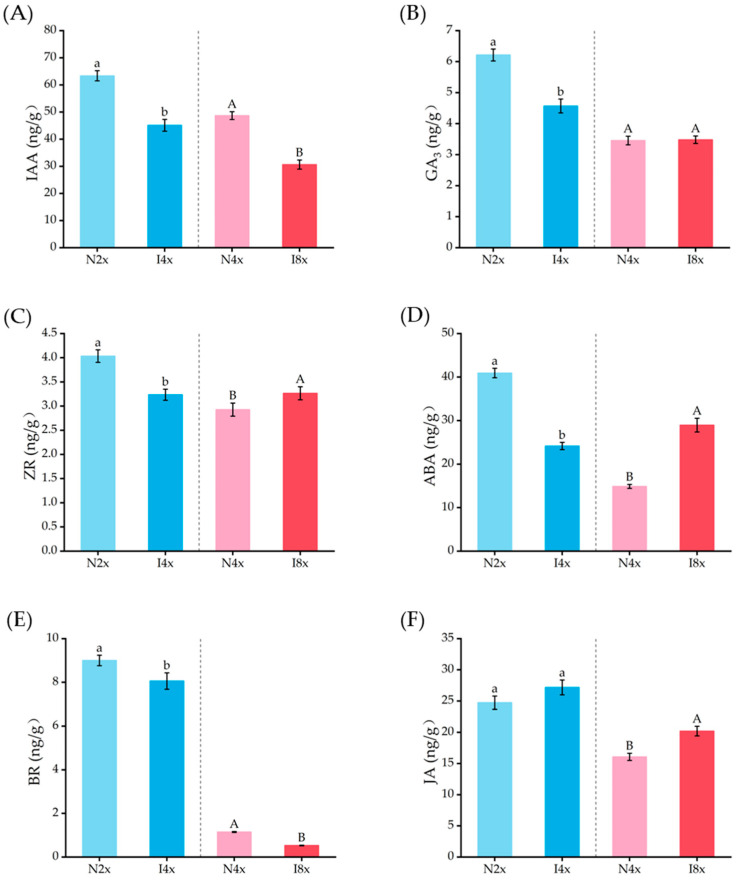
Comparison of six various phytohormone contents, including IAA (**A**), GA_3_ (**B**), ZR (**C**), ABA (**D**), BR (**E**), and JA (**F**), in leaves of different ploidies of *C. paliurus* seedlings. An independent-samples t-test was used to test the significance (*p* ≤ 0.05). Lowercase letters indicate significance between N2x and I4x (*p* ≤ 0.05), and uppercase letters indicate significance between N4x and I8x (*p* ≤ 0.05).

As indicated by Figure 7, the contents of six endogenous hormones (IAA, GA_3_, ZR, ABA, BR, and JA) were significantly affected by polyploidization, except for GA_3_ contents between I8x and N4x (*p* < 0.05). The IAA content decreased by 28.80% in I4x compared to N2x and decreased by 37.09% in I8x compared to N4x. In comparison to the control, the BR content in I4x was reduced by 10.5% and that in I8x decreased by 53.98%. Similarly, the ZR and GA_3_ contents in I4x decreased by 19.83% and 26.47%, respectively, compared to N2x, while the ZR content in I8x increased by 11.60% compared to N4x. With respect to the ABA content, I8x showed an increase of 94.70% compared to N4x. Furthermore, the JA content was increased by 9.85% in I4x and 25.79% in I8x compared to the controls.

## 3. Discussion

### 3.1. Effect of Colchicine on Polyploidy Induction

Colchicine is the most commonly used mutagen of polyploidy induction in plants, while the effectiveness of plant cell polyploidization can be accomplished by using an appropriate amount of colchicine and applying it for an optimal duration [15,16]. Moreover, optimal concentrations and treatment durations of colchicine for producing polyploidization varied among different plant species. For instance, fig trees treated with 625 μM colchicine for 48 h had the highest tetraploid induction rate [13], whereas *Populus hopeiensis* treated with 100 μM colchicine for 4 d had the highest octoploid induction rate [17]. *Rosa roxburghii* was treated with 20 mg/L colchicine for 15 d, which resulted in a maximum polyploid induction rate [18]. In this study, the highest polyploid induction in 2021 and in 2022 was achieved by applying 0.4% colchicine for 4 d on the apical meristematic tissues of cotyledon stage seedlings. According to Luo et al. [19], colchicine treatments with a 50% lethality rate are most effective. In this experiment, the plant death rate of the most effective induction protocol was approximately 50%, consistent with the findings of previous research.

### 3.2. Morphological and Anatomical Changes in Polyploidies

Most studies found that polyploid plants exhibit larger leaves, thicker stems, and an increased content of primary and secondary metabolites, as well as enhanced resistance [20,21]. However, a few studies found that the vegetative growth of some polyploids is lower than that of diploids, such as white poplar [22]. This study found that induced polyploids had significantly smaller compound leaf area, fewer compound leaves, and larger terminal leaflet length. Additionally, I8x had heavier specific leaf weight and thicker leaflet. These results are consistent with *Lycium chinense* [23] and *Celosia argentea* L. [24], which may be caused by the increased thickness of spongy and fenestrated tissues. In addition, seedling height and basal diameter of the induced polyploids were lower than those of the control in this study, consistent with the results of *Rhododendron fortunei* [12], *Populus hopeiensis* [17], and fireweed [25]. The reasons for the inhibition of plant growth by polyploidy are still controversial. For example, Manzoor et al. [26] pointed out that elevated levels of polyploidy may cause stunted plants due to somatic instability and extreme gene redundancy, while Sattler et al. [27] supposed that polyploids typically exhibit an increase in cell size, but the reduced number of cell divisions may result in low growth rates. Thus, it is necessary to regularly evaluate the stability of induced tetraploids and octaploids in future studies and to simultaneously observe the changes in phenotypic traits.

Stomatal dimensions, stomatal density, and the number of chloroplasts in stomata are often used as indications of polyploidy events, since these measurements are less affected by environmental factors [28]. Generally, stomatal length and width tend to increase concomitant with polyploidization, whereas stomatal density exhibits an inverse relationship [13,29]. A similar result was observed in this study, where the stomatal size of polyploid plants increased significantly, while stomatal density decreased sharply. In addition, the chloroplast counts of polyploid plants (I4x and I8x) were obviously higher compared to corresponding natural plants (N2x and N4x), in agreement with the results from *Lycium chinense* [23] and *Thymus vulgaris* [16]. Our results indicated that stomatal dimensions, stomatal density, and the number of chloroplasts could serve as reliable indicators for breeders to easily distinguish between polyploid and unpolyploid *C. paliurus*.

### 3.3. Biochemical Changes in Polyploidies

Besides physiological and anatomical traits, leaf phytochemical traits were obviously affected by polyploidy in plants. An increased content of carotenoids and chlorophyll is commonly reported during the induction of polyploids in several plant species [13,30]. Similarly, the induced polyploid plants generally exhibited higher levels of photosynthetic pigments compared to the control plants in our study. Correspondingly, their leaves showed increased contents of soluble sugars, starch, and soluble proteins. However, the number and area of compound leaves in induced polyploids significantly decreased. We supposed that the reduction in photosynthetic area resulted in less accumulation of carbon assimilation products and eventually inhibited the growth of polyploid *C. paliurus* (I4x and I8x). This phenomenon may be attributed to the overall increase in the contents of growth-inhibiting hormones (ABA and JA) and the decrease in the contents of growth-promoting hormones (such as IAA, ZR, GA3, and BR) in polyploid plants. As revealed by abundant studies, auxin, GAs, and BRs promote leaf growth by increasing cell proliferation and expansion [31], while cytokinins promote cell division and increase cell expansion during the proliferation and expansion stages of leaf cell development, respectively [32]. These results indicate that the effect of polyploidization on growth is achieved by regulating the hormone levels in *C. paliurus*.

In summary, an optimal polyploidy induction method was established based on systematic screening of colchicine concentration and treatment time. The stomatal length, stomatal width, stomatal density, and chloroplast number could be used as reliable indicators for breeders to determine whether *C. paliurus* has achieved polyploidy by selecting the indicators that have the same variation patterns in both diploid polyploidy and tetraploid polyploidy. In addition, the induced polyploids in this study had a dwarfing effect, which provides superior germplasm resources for leaf-harvesting plantation.

## 4. Materials and Methods

### 4.1. Plant Material

Seeds of JZS4x were obtained from Jinzhongshan (24°62′ N, 104°95′ E), Guangxi province, China, in November 2020, while seeds of WF2x and WF4x were collected in Wufeng, Hubei province, China, in late October 2021. The polyploidy of JZS4x and WF2x was diploid (2n = 4x = 32), while the polyploidy of WF4x was tetraploid (2n = 4x = 64). According to the method of Yang et al. [33], the seeds were subjected to exogenous GA3 (gibberellin A3) treatments and stratification treatments to promote seed germination. The germinated seeds were subsequently sown into nonwoven containers (8 cm in diameter and 11 cm in height), filled with the substrate (Jiangsu Xingnong Science and Technology Co., Ltd., Zhenjiang, China), and then moved into a greenhouse at Nanjing Forestry University.

### 4.2. Colchicine Treatment

The stem tip treatment method [34] was used for seedlings at the cotyledon stage. After the cotyledon unfolded and true leaves sprouted, the true leaves were cut off and then wrapped around the growth point of the stem tip with 0.01g of skimmed cotton containing colchicine (Shanghai Aladdin Biochemical Technology Co., Ltd., Shanghai, China) solution. A 2% (*V*/*V*) dimethyl sulfoxide (DMSO) (Shanghai Aladdin Biochemical Technology Co., Ltd., Shanghai, China) was added to the colchicine solution to improve the ability of cells to absorb colchicine. The colchicine solution was applied to the cotton twice a day (at 7:00 and 19:00), and the seedlings were shaded during treatment. After the treatment, the cotton was removed, and the residual solution was cleaned with water.

In 2021, colchicine solution concentration and treatment time were designed to investigate the effects of colchicine on polyploidization of *C. paliurus*. Seven treatments were used in this study, including 0.3% colchicine solution concentration for 4 d, 8 d, and 12 d, 0.4% colchicine solution concentration for 4 d and 7 d, and 0.6% colchicine solution concentration for 3d and 6d. Each treatment consisted of three replicates with 20 seedlings in each replicate. The seedlings were cultivated in the greenhouse with regular irrigation and uniform pest management. After 120 d, the survival and induction rates were calculated.

In 2022, WF4x seedlings were treated with 0.4% for 4 d to verify the feasibility of the colchicine treatment based on the experimental results from 2021. Each treatment was repeated three times with 16 seedlings in each replicate. Meanwhile, WF2x seedlings were treated with 0.4% for 4 d, 5 d, and 6 d. Each treatment was repeated three times with 28 seedlings in each replicate. After 150 d, the survival and induction rates were calculated and the successfully mutated seedlings (I4x and I8x) along with control seedlings (N2x and N4x) were used for further analyses.

### 4.3. Ploidy Identification

#### 4.3.1. Flow Cytometry (FCM) Analysis

The flow cytometry analysis was performed according to Song et al. [35], as follows: 1 mL of mGb dissociative solution (LEAGENE Biotechnology Co., Ltd., Huaibei, China) was added to a Petri dish on ice. Young leaves were finely chopped in the dissociative solution and then filtered through a 40 μm mesh filter into an EP tube to obtain the nucleus suspension. Afterward, 20 μL of propidium iodide (PI) dye (LEAGENE Biotechnology Ltd., Huaibei, China) was added to stain for 1 min at 4 °C in the dark. The sample tubes were loaded into the InfluxTM flow cytometer (BD, Franklin Lakes, NJ, USA) to determine their ploidy level.

#### 4.3.2. Chromosome Counting

Freshly curled young leaves were collected between 7:00 and 9:00 a.m. The samples were pretreated with saturated para-dichlorobenzene for 6 h at 4 °C and then placed in Carnoy’s fluid (ethanol/glacial acetic acid, 3:1, *v*:*v*) overnight. The fixed leaves were dissociated with a solution (ethanol and hydrochloric acid, 1:1, *v*:*v*) for 10 min at room temperature and then washed thrice with distilled water. After the leaves were stained with carbol fuchsin for 2 min and then transferred on a glass slide to squash using the conventional pressing method. Chromosome counting was performed using 1000× magnification of an Olympus BX 51 (Olympus Corporation, Tokyo, Japan).

### 4.4. Growth and Morphological Measurements

After the ploidy identification, the height and ground diameter of the mutated and control seedlings were measured. The fourth expanded mature compound leaf from the apical growing shoots of the standard plant was scanned using an Epson Expression 12000XL scanner (Seiko Epson Corporation, Nagano-ken, Japan), and then the data were analyzed using Adobe Photoshop CS6 for measuring compound leaf length, compound leaf width, petiole length, compound leaf area, number of leaflets, terminal leaflet length, terminal leaflet width, and terminal leaflet shape index.

### 4.5. Stomata Observation

Three standard plants from treated and non-treated (control) seedlings were chosen to observe stomatal characteristics. The lower epidermis layer of leaf samples (the third pair of leaflets from the third fully expanded leaves) was separated using nail varnish and sellotape. The stomata counting and density were estimated using a 200× magnification of an Olympus BX 51 (Olympus Corporation, Tokyo, Japan). A total of 15 randomly chosen fields of view per leaf and 10 stomata from each field of view were randomly selected to calculate mean values. For chloroplast counting, 0.1 cm^2^ of leaf was stained with a drop of 1% (*w*/*v*) potassium–potassium iodide solution for 5 min. The number of chloroplasts in the stomata was counted using an Olympus BX 51 (Olympus Corporation, Tokyo, Japan) at 400× magnification, with 100 stomata per leaf.

Stomatal density was calculated using the following formula:

$$D=\frac{n}{L*W_{z}}$$

n = number of stomata in the view;
L = length of view;
W = width of view.

### 4.6. Leaf Anatomical Structure Observation

A 0.5 cm^2^ piece was sampled from the leaf blade’s center in the second pair of leaflets at the third fully expanded leaf. Then, the sample was fixed using a formalin–acetic acid–alcohol (FAA) solution (Phygene Biotechnology Co., Ltd., Fuzhou, China) and processed through a standard paraffin sectioning procedure. The paraffin sections were examined and imaged under 200× magnification using an Olympus BX 51 microscope (Olympus Corporation, Tokyo, Japan).

### 4.7. Physiological and Biochemical Analysis

Nine seedlings per ploidy were randomly selected to collect the third fully expanded mature leaf per plant for the phytochemical analysis. The samples were immediately transferred to liquid nitrogen and then ground into a fine powder, preserved at −80 °C for subsequent analysis.

#### 4.7.1. Chlorophyll Content Measurement

The chlorophyll content was measured following the method of Wang et al. [36]. A total of 0.1 g frozen powder was mixed with 8 mL 95% ethanol and extracted for 24 h at room temperature in the dark. The mixtures were centrifuged at 4000 rpm for 10 min. The supernatants were measured at absorbances of 470 nm, 649 nm, and 665 nm.

#### 4.7.2. Primary Metabolite Content Measurement

Leaf total soluble sugars and starch were measured according to Wang et al. [36]. A total of 0.1 g of frozen powder was mixed with 15 mL of deionized water, followed by heating at 100 °C for 30 min. The supernatants were cooled and diluted to 100 mL. Then, 0.5 mL of the diluted supernatants was mixed with 1.5 mL of deionized water, 0.5 mL of Anthrone ethyl acetate reagent (1 g of anthrone + 50 mL of ethyl acetate), and 5 mL of sulfuric acid. The mixture was heated in a water bath at 100 °C for 1 min. After cooling, the absorbance was measured at 620 nm using a Specord 200plus spectrophotometer (Analytikjena, Jena, Germany) with sucrose as the standard. The remaining filter residue after extracting soluble sugars was mixed with 10 mL of deionized water, followed by heating in a water bath at 100 °C for 30 min. After the water bath, 2 mL of 9.2 mol/L perchloric acid was mixed and stood for 15 min. After the solution cooled to room temperature, the resulting mixture was diluted to 25 mL for measuring starch content. The absorbance was measured at 620 nm with starch as the standard.

Leaf total soluble proteins were determined according to Wang et al. [36]. A total of 0.5 g of frozen powder was homogenized with 5 mL of deionized water. The mixture was centrifuged at 3000 rpm at 4 °C for 10 min. Then, 0.2 mL of the supernatant was mixed with 5 mL of Coomassie Brilliant Blue solution, followed by standing for 5 min. The absorbance was measured at 595 nm with bovine serum protein as the standard.

#### 4.7.3. Endogenous Hormone Content Measurement

The endogenous levels of IAA, BR, GA_3_, JA, ABA, and ZR were determined following the method of Yang et al. [37]. A total of 1.0 g of the sample was homogenized in 4 mL of 80% methanol (containing 1 mmol/L butylated hydroxytoluene). The mixture was kept at 4 °C for 4 h and then centrifuged at 4 °C and 3500 rpm for 8 min. The supernatants were retained, and the precipitate was extracted again using the above method. The resulting supernatants were purified using a C-18 solid-phase extraction column. The purified solution was dried under nitrogen gas and then diluted to 2 mL with phosphate buffer solution (PBS, pH = 7.5), 0.5 mL Tween-20, and 0.5 g gelatin. A total of 5 mL of the diluted solution and the standard solution were mixed with antibodies on a scale of 2000:1, respectively, and then added to a 96-well plate. The 96-well plate was incubated at 37 °C for 0.5 h in a wet box. Then, the 96-well plate was washed four times using a detergent (1000 mL PBS plus 1 mL Tween-20). A total of 10 mL of the diluted solution was mixed with 10 μL of enzyme-linked secondary antibodies, and then 100 μL of the resulting mixture was added to the washed 96-well plate. Again, the 96-well plate was incubated at 37 °C for 0.5 h in a wet box and washed four times. The buffered enzyme substrate (o-phenylenediamine) was added into the 96-well plate, followed by conducting the enzyme reaction in the dark at 37 °C for 15 min. The reaction was terminated with 2 mol/L sulfuric acid. The absorbance was measured at 490 nm, and the hormone contents were calculated as Weiler et al. [38] described.

### 4.8. Statistical Analysis

Statistical analyses were conducted using Microsoft Excel 2019 and SPSS 22.0. Data were presented as mean ± standard deviation (SD). A comparison of the methods used in the colchicine treatments was performed utilizing one-way analysis of variance (ANOVA) and Duncan’s multiple range tests. An independent-samples t-test was performed to assess differences in indexes between natural plants and induced plants (*p* < 0.05). Histograms were generated using Origin 2021.

## 5. Conclusions

This study established an efficient polyploid induction method for *C. paliurus*. Based on the survival rate and polyploid induction rate, the optimal treatment condition was 0.4% colchicine for 4 d. Through FCM analysis and chromosome counting, 40 induced tetraploid plants and 33 induced octoploid plants were obtained from all treatment groups. Compared to natural plants, the induced polyploid plants exhibited regulated morphology, physiology, and biological characteristics, including a reduced number of compound leaves, reduced compound leaf area, enlarged compound terminal leaves, expanded stomata, more chloroplasts, increased contents of starch, soluble sugars, and soluble proteins, increased contents of growth-inhibiting hormone, and decreased contents of growth-promoting hormone. Since *C. paliurus* is endemic to China, our results have a rather local effect. Overall, the results of this study provide technical support for the polyploid breeding of *C. paliurus*. Moreover, the induced polyploid plants with a dwarfing effect have the potential to be used as superior genetic resources for leaf harvesting.

## Figures and Tables

**Table 1:** Effects of different colchicine concentrations and treatment time on induction of *C. paliurus* seedlings.

Year	Provenance	Colchicine Concentration (%)	Time of Treatment (d)	Number of Treated Explants	Survival Rate (%)	Polyploid Induction Rate (%)	Mixoploid Induction Rate (%)
2021	JZS4x	0.3	4	60	5.00 ± 5.00 b	1.67 ± 2.87 b	1.67 ± 2.87 b
		0.3	8	60	6.67 ± 2.87 b	0.00 ± 0.00 b	5.00 ± 5.00 b
		0.3	12	60	8.33 ± 2.87 b	1.67 ± 2.87 b	5.00 ± 0.00 b
		0.4	4	60	35.00 ± 13.23 a	20.00 ± 5.00 a	10.00 ± 5.00 a
		0.4	7	60	1.67 ± 2.87 b	0.00 ± 0.00 b	0.00 ± 0.00 b
		0.6	3	60	0.00 ± 0.00 b	0.00 ± 0.00 b	0.00 ± 0.00 b
		0.6	6	60	0.00 ± 0.00 b	0.00 ± 0.00 b	0.00 ± 0.00 b
2022	WF4x	0.4	4	48	52.08 ± 7.80	39.58 ± 7.80	8.33 ± 2.95
	WF2x	0.4	4	84	51.19 ± 4.12 a	33.33 ± 7.43 a	5.95 ± 5.46 a
		0.4	5	84	19.05 ± 2.06 b	14.29 ± 3.57 b	4.76 ± 2.06 a
		0.4	6	84	2.38 ± 4.12 c	0.00 ± 0.00 c	2.38 ± 4.12 a

Data are presented as mean ± SD (standard deviation). Duncan’s multiple test was used to test the significance. Lowercase letters indicate significance among treatments (*p* ≤ 0.05).

**Table 2:** Differences in morphological traits between different ploidy levels of *C. paliurus* seedlings.

Characteristics	N2x	I4x	N4x	I8x
Seedling height (cm)	31.57 ± 0.10 a	25.67 ± 0.06 b	33.92 ±0.12 A	31.17 ± 1.81 A
Basal diameter (mm)	6.08 ± 0.04 a	5.28 ± 0.34 a	6.31 ±0.04 A	5.15 ± 0.22 B
Compound leaf area (cm^2^)	116.73 ± 5.01 a	95.93 ± 10.73 b	144.3 ± 2.71 A	75.43 ± 1.80 B
Specific leaf weight (g/m^2^)	22.60 ± 0.72 a	28.83 ± 4.89 a	25.07 ± 0.83 B	27.08 ± 0.79 A
Number of compound leaves	9.67 ± 1.53 a	6.33 ± 0.58 b	9.33 ± 0.58 A	6.67 ± 1.15 B
Terminal leaflet length (cm)	7.72 ± 0.25 b	9.22 ± 0.81 a	8.41 ± 0.02 B	10.69 ± 1.30 A
Terminal leaflet width (cm)	2.90 ± 0.20 a	3.44 ± 0.71 a	3.59 ± 0.36 B	4.53 ± 0.37 A

Data is presented as mean ± SD (standard deviation). An independent-samples *t*-test was used to test the significance (*p* ≤ 0.05). Lowercase letters indicate significance between N2x and I4x (*p* ≤ 0.05), and uppercase letters indicate significance between N4x and I8x (*p* ≤ 0.05).

**Table 3:** Differences in leaf blade structure between different ploidy levels of *C. paliurus* seedlings.

Characteristics	N2x	I4x	N4x	I8x
Leaflet thickness (μm)	83.84 ± 3.64 a	89.02 ± 1.32 a	92.84 ± 0.66 B	110.50 ± 1.33 A
Upper epidermal thickness (μm)	11.15 ± 1.70 a	10.40 ± 0.72 a	12.12 ± 1.05 A	11.20 ± 0.83 A
Lower epidermal thickness (μm)	10.07 ± 1.13 a	11.05 ± 1.73 a	11.68 ± 0.61 A	11.12 ± 0.85 A
Palisade tissue thickness (μm)	31.18 ± 2.74 a	35.48 ± 0.80 a	36.44 ± 2.45 B	43.32 ± 1.04 A
Spongy tissue thickness (μm)	31.44 ± 2.25 a	32.09 ± 1.68 a	32.60 ± 2.18 B	44.86 ± 1.11 A

Data is presented as mean ± SD (standard deviation). An independent-samples *t*-test was used to test the significance (*p* ≤ 0.05). Lowercase letters indicate significance between N2x and I4x, and uppercase letters indicate significance between N4x and I8x.

**Table 4:** Differences in stomatal characteristics between different ploidy levels of *C. paliurus* seedlings.

Characteristics	N2x	I4x	N4x	I8x
Stomatal length (μm)	25.23 ± 2.40 b	36.00 ± 1.36 a	30.68 ± 2.50 B	35.57 ± 0.80 A
Stomatal width (μm)	17.64 ± 1.87 b	22.49 ± 2.33 a	20.42 ± 1.91 B	24.74 ± 3.36 A
Stomatal density (N/mm^2^)	156.99 ± 3.75 a	57.41 ± 1.06 b	126.93 ± 10.31 A	50.01 ± 1.35 B
Chloroplast number (N/Stoma)	14.52 ± 0.52 b	21.42 ± 2.11 a	23.04 ± 2.06 B	30.24 ± 0.81 A

Data is presented as mean ± SD (standard deviation). An independent-samples *t*-test was used to test the significance (*p* ≤ 0.05). Lowercase letters indicate significance between N2x and I4x (*p* ≤ 0.05), and uppercase letters indicate significance between N4x and I8x (*p* ≤ 0.05).

## Data Availability

The original contributions presented in this study are included in the article. Further inquiries can be directed to the corresponding author.

## Abbreviations

The following abbreviations are used in this manuscript:

N2x	Natural diploid
I4x	Induced tetraploid
N4x	Natural tetraploid
I8x	Induced octoploid
ZR	Zeatin riboside
IAA	Indole-3-acetic acid
BR	Brassinosteroids
GA_3_	Gibberellic acid
JA	Jasmonic acid
ABA	Abscisic acid
